# Coronin 2A (CRN5) expression is associated with colorectal adenoma-adenocarcinoma sequence and oncogenic signalling

**DOI:** 10.1186/s12885-015-1645-7

**Published:** 2015-09-15

**Authors:** Raphael H. Rastetter, Margit Blömacher, Uta Drebber, Marija Marko, Juliane Behrens, Roxana Solga, Sarah Hojeili, Kurchi Bhattacharya, Claudia M. Wunderlich, F. Thomas Wunderlich, Margarete Odenthal, Anja Ziemann, Ludwig Eichinger, Christoph S. Clemen

**Affiliations:** 1 Center for Biochemistry, Institute of Biochemistry I, Medical Faculty, University of Cologne, Joseph-Stelzmann-Street 52, 50931 Cologne, Germany; 2 Institute of Pathology, University Hospital of Cologne, 50931 Cologne, Germany; 3 Institute for Genetics, University of Cologne, 50674 Cologne, Germany; 4 Max Planck Institute for Neurological Research, 50931 Cologne, Germany; 5 Present address: Department of Anatomy and Developmental Biology, Monash University, Clayton, VIC 3800 Australia

## Abstract

**Background:**

Coronin proteins are known as regulators of actin-based cellular processes, and some of them are associated with the malignant progression of human cancer. Here, we show that expression of coronin 2A is up-regulated in human colon carcinoma.

**Methods:**

This study included 26 human colon tumour specimens and 9 normal controls. Expression and localisation of coronin 2A was studied by immunohistochemistry, immunofluorescence imaging, cell fractionation, and immunoblotting. Functional roles of coronin 2A were analysed by over-expression and knock-down of the protein. Protein interactions were studied by co-immunoprecipitation and pull-down experiments, mass spectrometry analyses, and *in vitro* kinase and methylation assays.

**Results:**

Histopathological investigation revealed that the expression of coronin 2A in colon tumour cells is up-regulated during the adenoma-adenocarcinoma progression. At the subcellular level, coronin 2A localised to multiple compartments, i.e. F-actin stress fibres, the front of lamellipodia, focal adhesions, and the nuclei. Over-expression of coronin 2A led to a reduction of F-actin stress fibres and elevated cell migration velocity. We identified two novel direct coronin 2A interaction partners. The interaction of coronin 2A with MAPK14 (mitogen activated protein kinase 14 or MAP kinase p38α) led to phosphorylation of coronin 2A and also to activation of the MAPK14 pathway. Moreover, coronin 2A interacted with PRMT5 (protein arginine N-methyltransferase 5), which modulates the sensitivity of tumour cells to TRAIL-induced cell death.

**Conclusions:**

We show that increased expression of coronin 2A is associated with the malignant phenotype of human colon carcinoma. Moreover, we linked coronin 2A to MAPK14 and PRMT5 signalling pathways involved in tumour progression.

**Electronic supplementary material:**

The online version of this article (doi:10.1186/s12885-015-1645-7) contains supplementary material, which is available to authorized users.

## Background

Colon cancer is one of the most frequent cancers [1] and its progression is classified into five stages, in which in stage 0 the cancer involves only the mucosa and in stage IV the cancer has spread to a distant organ or set of distant lymph nodes [2]. The majority of colon cancers arise from pre-malignant adenomas. Although new screening methods and early diagnosis have increased survival rates in the past few years, the mortality rates of patients diagnosed with one of the later stages are still very high [3]. Once colon cancer has reached disease stage IV the five-year survival rate drops to 8.1 % [4]. It is therefore of major interest to identify cellular pathways involved in the migration and invasion of colon tumour cells.

Coronin proteins belong to the superfamily of eukaryotic-specific WD40-repeat domain proteins [5]. They play important roles in the regulation of F-actin dynamics in numerous cellular processes including the migration and invasion of tumour cells [6]. Phylogenetic analyses of the coronin family of proteins defined seventeen coronin subfamilies including seven paralogs in mammals [7–9]. Mammalian coronin 2A (synonyms are coronin 4, ClipinB, IR10, and CRN5; the latter is used in this study) is a member of the ‘short’ coronin subfamily containing a single WD40-repeat domain, which adopts the fold of a seven bladed β-propeller [10]. Compared to other well-characterized coronins less information is available about CRN5. In rat mammary adenocarcinoma cells a knock-down of CRN5 led to reduced cell migration velocity and increased size, decreased number, and decreased disassembly of focal-adhesions. Based on the observations that CRN5 interacts with the cofilin-activating phosphatase Slingshot-1 L and knock-down of CRN5 increases the amount of phospho-cofilin, CRN5 has been implicated in the regulation of the focal adhesion turnover rate [11]. In addition, CRN5 has been identified as a component of the nuclear receptor co-repressor (NCoR) complex [12] with a function as an NCoR exchange factor [13]. Here, the interaction of CRN5 via a SIM-motif (small ubiquitin-like modifier (SUMO) 2/3 interacting motif) located in its coiled coil region with SUMOylated liver X receptors (LXRs) prevents NCoR clearance from target gene promoters. In absence of SUMOylated LXRs CRN5 binds to oligomeric nuclear actin enabling NCoR clearance and de-repression of Toll-like receptor-induced inflammatory response genes in macrophages [13].

The protein kinase MAPK14 is stimulated by pro-inflammatory signals and environmental stresses such as heat shock, irradiation, and ultraviolet light leading to its activation via phosphorylation at Thr180 and Tyr182 by upstream MAPK kinases 3 and 6 [14]. An involvement of MAPK14 in colorectal cancer has been shown in Apc^Min^ colorectal cancer mice, which showed a significant reduction in tumour size when they were treated with the MAPK14 inhibitor SB202190 after azoxymethane induction of colon tumours [15]. However, ablation of MAPK14 in the epithelial cells of the digestive tract of another mouse strain caused development of significantly more tumours [16]. In several human colon cancer cell lines the inhibition of MAPK14 by SB202190 induced growth arrest and autophagic cell death [17].

Protein arginine methyltransferases are important regulators of chromatin structure and gene expression, but are also involved in other cellular processes. PRMT5, a type II protein arginine methyltransferase enzyme, turned out to play a role in malignant transformation [18]. Moreover, PRMT5 functions in growth-promoting and pro-survival signalling pathways. Here, it has been reported that a knock-down of PRMT5 restored the sensitivity of several tumour cell lines to TRAIL-induced cell death [19].

In the present study, we demonstrate that the expression level of CRN5 is associated with the malignant progression of colon carcinoma, and that CRN5 over-expression led to elevated tumour cell migration velocity as well as altered MAPK14 and PRMT5 signalling pathways.

## Methods

### Immunohistochemistry

Tissue sections of paraffin-embedded human colon specimens comprised six low grade adenoma, five high grade adenoma, seven adenocarcinoma, five liver metastases, three lymph node metastases, six normal colon controls, and three normal (non-affected) lymph nodes. The normal colon controls were prepared from adjacent normal tissue of the surgical tissue specimens. After epitope retrieval using pre-heated sodium citrate buffer, the sections were immunolabelled with the mouse monoclonal antibody K77-578-1 directed against human CRN5. This was followed by blocking of biotin using the Avidin-Biotin Blocking Kit (Vector Laboratories). The primary antibody was detected by a biotin-conjugated rabbit anti-mouse secondary antibody (1:200 for 30 min; DAKO). Subsequently, avidin-conjugated horseradish peroxidase (Vector Laboratories) was applied following the manufacturer’s instructions and visualized by 10 min 3-amino-9-ethylcarbazole (AEC) chromogen (DAKO) incubation. Sections were then counterstained with haematoxylin (DAKO), dehydrated, and mounted. The use of these human tissue specimens for protein expression studies was approved by the local ethics committee of the University Hospital of Cologne, informed consent was obtained from the patients, and the specimens were procured from the centralized Biobank of the Center for Integrated Oncology (CIO) Cologne-Bonn (http://www.cio-koeln-bonn.de/en/medical-professionals/biobank/).

### Cell lines

A7r5 (ATCC CRL-1444) rat smooth muscle cells, CMT93 mouse rectum carcinoma cells (ECACC 89111413), CT26 mouse colon carcinoma cells (ATCC CRL-2638), DLD-1 and WiDr human colon carcinoma cells (ECACC 90102540 and 85111501), DHD/K12/TRb rat colonic carcinoma cells (ECACC 90062901), HEK293 human embryonic kidney cells (ATCC CRL-1573) and the variant 293TN pseudoviral particle producer cells (BioCat/SBI: LV900A-1), HeLa human cervix carcinoma cells (ATCC CCL-2), and U373 human glioblastoma cells (ECACC 89081403) were grown in the recommended nutrition media at 5 % CO_2_ and 37 °C. The rationale for using a particular cell line is given in the Results section.

### Antibodies

The following antibodies were used to detect CRN5, mouse mAb K77-578-1 (newly generated for the purpose of this study; affinity purified from hybridoma supernatant; dilution 1:500 in 4 % milk powder/PBS for western blotting, and 1:100 in 1 % FBS/PBS for immunofluorescence and immunohistochemistry), rabbit pAb H-110 (SantaCruz sc-66841, 1:500 in 5 % milk powder/TBS-T), mouse mAb A-3 (SantaCruz sc-271873, 1:250 in TBS-T buffer), and goat pAb L-20 (SantaCruz sc-32203, 1:250 in 5 % milk powder/TBS-T). Note that among the purchased CRN5 antibodies pAb H-110 worked best for human cell lysates and recombinant CRN5, mAb A-3 for mouse cell lines and tissues, and pAb L-20 for human cell lines and tissues.

β-actin was detected with mouse mAb AC-74 (Sigma A5316, 1:10,000 in TBS-T), Arp2/3-complex with rabbit pAb anti-p34 (Upstate 07-227, 1:400 in PBS), GAPDH by a mouse mAb (Sigma G9295, 1:10,000 in PBS), GFP with mouse mAb K3-167-2 ([20]; for immunoprecipitation), GST with a rabbit pAb ([21]; 1:50,000 in TBS-T), lamin B1 with a rabbit pAb (Abcam ab16048, 1:3,000 in TBS-T), PRMT5 with mouse mAb PRMT5-21 (Sigma P0493, 1:1,000 in in TBS-T), α-tubulin with rat mAb YL1/2 ([22], 1:10 in TBS-T), and vinculin with a rabbit pAb (Sigma V4139, 1:1,000 in 1 % FBS/PBS).

The following antibodies were used to analyse the MAPK14 pathway (all from Cell Signaling/New England Biolabs): phospho-MAPK14 (Thr180/Tyr182) rabbit mAb D3F9 (#4511, 1:1,000 in 5 % BSA/TBS-T), total MAPK14 rabbit pAb (#9212, 1:1,000 in 5 % BSA/TBS-T for western blotting and 1:100 in in 5 % BSA/PBS for immunofluorescence), phospho-MAPKAPK2 (Thr334) rabbit mAb (27B7) (#3007, 1:1,000 in 5 % BSA/TBST), total MAPKAPK2 rabbit pAb (#3042, 1:1,000 in 5 % BSA/TBS-T), phospho-Hsp27 (Ser82) rabbit mAb D8A8 (#2401, 1:1,000 in 5 % BSA/TBS-T), total Hsp27 mouse mAb G31 (#2402, 1:1,000 in 5 % milk powder/TBS-T), phospho-ATF2 (Thr71) rabbit pAb (#9221, 1:1,000 in 5 % BSA/TBS-T), total ATF2 rabbit mAb 20 F1 (#9226, 1:1,000 in 5 % BSA/TBS-T), phospho-p53 (Ser392) rabbit pAb (#9281, 1:1,000 in 5 % BSA/TBS-T), and total p53 mouse mAb 1C12 (#2524, 1:1,000 in 5 % milk powder/TBS-T).

### Immunoblotting and immunofluorescence imaging

For immunoblotting, protein samples were separated by 10 %, 12 % or 15 % SDS-PAGE under reducing conditions and proteins were transferred onto PVDF membranes by the wet-blotting method [23].

For immunofluorescence imaging cells were grown on 12 mm coverslips, washed with PBS, fixed in 4 % paraformaldehyde/PBS for 20 min at room temperature, permeabilized with 0.5 % Triton X-100 or 0.2 % saponin in PBS (identical results with both permeabilisation protocols), incubated in 0.15 % glycine/PBS for 10 min, washed three times with 0.05 % Triton X-100 or 0.02 % saponin in PBS, and blocked in 1 % FBS/PBS for 30 min. Incubation with the primary antibody was done in 1 % FBS/PBS for two hours, followed by three washes with PBS for five minutes each, incubation with secondary antibody AlexaFluor-488 or -568 diluted 1:500 in 1 % FBS/PBS for one hour, and three more washes with PBS for five minutes each. Finally, coverslips were rinsed in water and embedded in Gelvatol [24]. In order to visualise CRN5 in nuclei, cells were treated with 0.05 % Trypsin for 10 min for epitope retrieval [25] before proceeding to blocking in 1 % FBS/PBS.

### Cell migration assays

Cell migration was measured using μ-slide 8-well chambers with silicone culture-inserts (ibidi #80209, #80826). 40,000 cells were seeded in each chamber and grown for 24 h. After removal of the culture-inserts, which leaves a 500 μm cell-free gap, images were taken every 15 min for a period of 20 h. Single cell tracking was done using the “Manual Tracking” (Fabrice Cordelières, Institut Curie, Orsay, France) plugin and cell path were analysed using the “Chemotaxis and Migration Tool” (ibidi) plugin for ImageJ (NIH). Directionality is calculated by the Euclidian divided by the accumulated distance.

### Cell fractionation

Purification of nuclei was performed according to [26]. In brief, washed cell pellets were re-suspended in ice-cold PBS containing 0.1 % NP-40, and, after lysis, separated by centrifugation into a cytosolic supernatant and a pellet of intact nuclei; the latter was washed and centrifuged a second time before further use.

### CRN5-related plasmids

In order to change the cellular expression level of CRN5, two plasmids for lentiviral transduction were newly generated. The first, pLKO.1-CRN5-shRNA-puro, was used for shRNA-mediated knock-down of murine CRN5. It was generated by annealing oligos CCGGGAGGGAACGTCTTGGACATCTCGAGATGTCCAAGACGTTCCCTCTTTTTT (forward, with adjacent AgeI restriction site overhang) and AATTCAAAAAAGAGGGAACGTCTTGGACATCTCGAGATGTCCAAGACGTTCCCTC (reverse, with adjacent EcoRI restriction site overhang), and cloning of the resulting duplex into AgeI/EcoRI opened pLKO.1-puro. The CRN5 target sequence of the expressed shRNA is GGAACGTCTTGGACATCAT. pLKO.1-nontarget-shRNA-puro [27] expressing a non-target shRNA was used for control experiments. The second, pLKO.1-CMV-EGFP-CRN5-puro, was used for over-expression of GFP-CRN5. For its generation, a CRN5 PCR product with adjacent EcoRI sites (primer pair: TCGAATTCCTGCAGGTATACGATATCAATGTCATGGCACCCCCAGTACCGGAGC and GAGAATTCTTATTAATGATCATCTAGACCCGGGGTCGACGATATCTCAGAGCTGCTCTGAGCCCATCCG) was amplified from pCMV6-XL4 carrying the human CRN5 cDNA NM_052820 (origene SC109892) and inserted into pLKO.1-CMV-EGFP-MCS-puro [28]. pLKO.1-CMV-EGFP-puro [28] expressing GFP alone was used as the corresponding control.

### Cell transfection and transduction

Transient transfections of A7r5 and U373 cells were performed by electroporation (Amaxa/Lonza Nucleofector II, Cell Line Nucleofector Solution V, programs X-001 (A7r5) and T-020 (U373)) and of HEK293 and HeLa cells by lipid-mediated transfection using Lipofectamine 2000 (Invitrogen). HEK293 cells stably expressing GFP or GFP-CRN5 were generated by neomycin (G418) selection.

Lentiviral transduction of CT26 and U373 cells was performed as described in [27]. In brief, each plasmid of interest was mixed with three lentiviral packaging plasmids and co-transfected into 293TN cells. Resulting lentiviruses were harvested and used to transduce the target CT26 and U373 cells. After transduction CT26 and U373 cells were further enriched by addition of 9.00 μg/ml and 0.75 μg/ml puromycine to the growth medium, respectively.

### Microscopy

Immunohistochemistry images were recorded with a Olympus microscope equipped with the photoP software, immunofluorescence images with a Leica TCS SP5/AOBS/tandem scanning system with emission detection in sequential mode equipped with the Leica LAS-AF software (v. 2.6.0.7266), and cell migration images with a Leica DMI6000B TIRF MC system equipped with software LAS-AF (v. 2.0.2.2038) and Hamamatsu EM-CCD camera C9100-02 in bright field (TL-PH) mode.

### Affinity purification of tagged proteins from *E. coli*

GST-CRN5 (above CRN5 cDNA cloned into pGEX-4 T-1), GST-MAPK14 (MAPK14 cDNA L35264.1 in pReceiver-B03, GeneCopoeia EX-A1099-B03), and GST (empty pGEX-4 T-1) were expressed in *E. coli* strain BL21. Protein expression was induced at an OD_600_ of 0.6-0.8 with 0.5-1 mM IPTG for 2-4 h at room temperature or 37 °C. Cells were pelleted at 5,000 g for five minutes, re-suspended in lysis buffer (50 mM Tris/HCl pH 8.0, 150 mM NaCl, 0.5 % Triton X-100, 2 mM EDTA, 2 mM EGTA, 4 mM DTT, 0.5 mM PMSF, 10 μg/ml aprotinin, 10 μg/ml leupeptin, 2 mM benzamidin, 100 μg/ml lysozyme) for 15 min at 4 °C, snap frozen two times in liquid nitrogen, and sonicated for two minutes. After centrifugation of the lysates for 30 min at 13,000 g and 4 °C, the supernatants were incubated with glutathione-beads for 2-4 h at 4 °C, and beads were washed 4-8 times in 20 mM Tris/HCl pH 8.0, 300 mM NaCl. GST-fusion proteins were eluted with 20 mM glutathione, 200 mM Tris/HCl pH 8.0, 2 mM EGTA, 0.1 % Triton X-100. For purification of untagged MAPK14, beads were incubated with the TEV protease (enzyme with both GST- and His-tags, Sigma T4455) in 50 mM Tris/HCl pH 8.0, 150 mM NaCl, 0.5 % Triton X-100.

For purification of His6-PRMT5 (cDNA AF167572.1 in pReceiver-B01, GeneCopoeia EX-V1247-B01) cells were lysed in 150 mM Tris/HCl, pH 8.0, 300 mM NaCl, 40 mM imidazole, 0.5 % Triton X-100, 5 % glycerol, 4 mM DTT, 0.5 mM PMSF, 10 μg/ml aprotinin, 10 μg/ml leupeptin, 2 mM benzamidin, 150 μg/ml lysozyme. Ni-beads were washed with 150 mM Tris/HCl, pH 8.0, 300 mM NaCl, 40 mM imidazole, 0.5 % Triton X-100, 5 % glycerol, and eluted in 150 mM Tris/HCl, pH 8.0, 150 mM NaCl, 250 mM imidazole.

### Affinity purification of tagged proteins from mammalian cells

GFP-CRN5 (above CRN5 cDNA cloned into pEGFP-C1) and GST-MAPK14 (MAPK14 cDNA L35264.1 in pReceiver-M04, GeneCopoeia EX-A1099-M04) were over-expressed in HEK293 cells. Cells were scraped off the culture dishes, washed once with PBS, and lysed in 50 mM Tris/HCl pH 8.0, 150 mM NaCl, 0.5 % Triton X-100, 2 mM EDTA, 2 mM EGTA, 4 mM DTT, 0.5 mM PMSF, 10 μg/ml aprotinin, 10 μg/ml leupeptin, 2 mM benzamidin. Supernatants were incubated with GFP-antibody coated (Miltenyi #130-091-125) or GST-antibody coated microbeads (Miltenyi #130-091-370) for two hours at 4 °C. The mixture was passed through μ Columns (Miltenyi), which had been rinsed with 200 μl of 50 mM Tris HCl pH 8.0, 150 mM NaCl, 1 % NP-40, 0.5 % sodium deoxycholate, 0.1 % SDS, using the Octomacs Separator (Miltenyi). After four additional washes with 200 μl of this buffer with addition of 5 mM Mg-ATP and one further wash with 100 μl of 20 mM Tris HCl pH 7.5, the proteins were eluted by a pH shift; 100 μl 0.1 M triethylamine pH 11 were added to the columns, and eluates were collected in tubes containing 13 μl 1 M MES pH 3 for neutralisation.

### CRN5 co-immunoprecipitation and pull-down assays

For co-immunoprecipitation lysates of *E. coli* expressing GST-CRN5 were incubated with 5-10 μg primary antibody K77-578-1 (anti-CRN5) and 50 μl Protein G-coated magnetic microbeads (Miltenyi #130-071-101) for two hours at 4 °C. The mixture was passed through μ Columns/Octomacs Separator and the columns were washed five times as above. Next, above GST-MAPK14 purified from HEK293 cells or GST protein from *E. coli* were circulated five times through the columns before the columns were again washed five times (4× 200 μl/1× 100 μl) and eluted with 120 μl of pre-heated SDS-PAGE sample buffer 50 mM Tris HCl pH 6.8, 50 mM DTT, 1 % SDS, 1 mM EDTA, 0.005 % bromphenol blue, 10 % glycerol.

For pull-down assays glutathione-beads carrying GST-CRN5 or GST as baits were incubated in 50 mM Tris/HCl pH 8.0, 150 mM NaCl, 0.5 % NP-40 with either TEV-protease tag-cleaved MAPK14 or purified His6-PRMT5 as prey, washed three times with the same buffer and analysed by SDS-PAGE.

### CRN5 immunoprecipitation and co-immunoprecipitation in conjunction with mass spectrometry

For CRN5 immunoprecipitation and analysis of post-translational modifications, U373 cells stably over-expressing GFP-CRN5 were scraped off, washed with PBS, and re-suspended in 50 mM Tris/HCl pH 8.0, 150 mM NaCl, 1 % NP-40, 2 mM EDTA, 0.5 mM PMSF, 2 mM benzamidin, 10 μg/ml leupeptin, 10 μg/ml aprotinin, 5 μM pepstatin A. Cells were homogenised by a tight douncer (five strokes) and five passages through a 21G needle. After centrifugation of the cell lysates for 15 min at 15,000 g and 4 °C, supernatants were incubated with 5-10 μg CRN5 antibody K77-578-1 and 50 μl Protein G coated sepharose beads for two hours at 4 °C. Beads were washed five times in PBS/1 % NP-40 and boiled in SDS sample buffer.

For CRN5 co-immunoprecipitation and detection of novel binding partners U373 cells stably over-expressing GFP-CRN5 were used with GFP-antibody coated microbeads as described above. Washing steps were done with 50 mM Tris HCl pH 8.0, 150 mM NaCl, 0.1 % Triton X-100, and proteins were eluted with pre-heated SDS-PAGE sample buffer.

Mass spectrometry was performed as described in [29]. In brief, proteins were separated by SDS-PAGE, stained with Coomassie brilliant blue, and bands were excised and proteins were identified after in-gel digestion with V8 or trypsin protease by liquid chromatography tandem-mass spectrometry employing a HCT ETD II iontrap mass spectrometer equipped with a nano ESI source (Bruker Daltonics, Bremen, Germany). Putative phosphopeptides (neutral loss of H_3_PO_4_) reported by Mascot searches (“Phosphorylation (ST)” was used as variable modification) were verified by manual inspection of the corresponding MS2 spectra.

### *In vitro* kinase assay

Kinase reactions were carried out in 40 μl volume containing 0.2 μg GST-MAPK14, 1 μg GFP-CRN5, 5 μl 8× kinase buffer (400 mM HEPES pH 8.0, 160 mM MgCl_2_, 40 mM EGTA, 160 mM β-glycerol phosphate, 8 mM Na_3_OV_4_, 8 mM DTT), and started by addition of 3 μl ATP mix (0.2 mM ATP, 0.4 μCi/μl γ^32^P-ATP (GE Healthcare)). Reaction times vary from 10 to 90 min at 32 °C and were stopped by adding 8 μl of 5× SDS-PAGE sample buffer. 30 μl of each experiment were resolved by SDS-PAGE, gels were Coomassie brilliant blue stained, dried and exposed to Kodak X-Ray films for 72 h. Controls were without GFP-CRN5 or GST-MAPK14, or in the presence of 92.5 μM MAPK14 inhibitor SB202190.

### Analysis of the MAPK14 signalling pathway

200,000 HEK293 cells over-expressing GFP-CRN5 or GFP were seeded in 6-well plates, grown overnight, exposed to heat shock at 42 °C for 0, 15, and 30 min according to [30], washed with PBS, and finally lysed in SDS-PAGE sample buffer for immunoblotting analyses. Cells were counted using a TC10TM automated cell counter (Bio-Rad Laboratories GmbH); GAPDH was used as a loading control.

### *In vitro* methylation assay

10 μl (350 ng) recombinant FLAG-PRMT5 in a complex with its activator His6-MEP50 purified from insect cells (BPS Bioscience # 51048; buffer was exchanged to 0.1 M Tris pH 8.0 using illustra NAP-5 gel filtration columns (GE Healthcare)), 20 μl (1.5 μg) full-length GFP-CRN5 purified from mammalian HEK293 cells (see above), which were treated with 20 mM adenosine dialdehyde (AdOx, inhibitor of S-adenosylhomocysteine nucleosidase) for 24 h before purification, or control substrates or elution buffer (no substrate), 5 μl ^3^H-labelled S-adenosylmethionine (PerkinElmer NET155, 0.55 mCi/ml, 15 Ci/mmol), and 0.1 M Tris pH 8.0 up to a final reaction volume of 57 μl were incubated for 4 h at 37 °C. Subsequently, proteins were methanol/chloroform precipitated and washed, solubilised in 100 μl 0.1 M HCl, 2 % SDS, and mixed with 4 ml Quicksafe A (Zinsser Analytic) to measure the ^3^H disintegrations per minute (dpm) in a scintillation counter (Wallac 1400 DSA version 2.4; protocol “easy count”, counting time 60 s).

### TRAIL resistance assay

HeLa cells were seeded in 6-well plates, transiently double-transfected on the next day at 30 % confluence with the following combinations of expression plasmids: empty pLKO.1-puro/empty pCMV-3xFLAG-6, PRMT5-shRNA oligo gatggacaatctggaatct (targets mouse and human PRMT5; GeneCopoeia MSH031088-HIVmU6-3-OS304859)/empty pCMV-3xFLAG-6, empty pLKO.1-puro/FLAG-CRN5 (above CRN5 cDNA cloned into pCMV-3xFLAG-6), and PRMT5-shRNA/FLAG-CRN5. 24 h after transfection growth medium was exchanged and one set of transfected cells received 50 ng/ml human TRAIL ligand (Enzo Life Sciences, SuperKillerTRAIL, ALX-201-115). After incubation for further 24 h cell proliferation rates were determined with the Quick cell proliferation assay Kit II (BioVision) employing the Tecan Infinite M1000 plate reader (absorbance values at 440 nm (dye) and at 650 nm (control/reference), multiple positions per well, measurements 0, 0.5, 1.0, 1.5, 2.0, 2.5, 3.0 h after addition of the dye reagent) before the cells finally were harvested for SDS-PAGE and immunoblotting.

### Data analysis and figure preparation

Data analyses and statistical evaluations were performed using Excel 2010 (Microsoft); column charts indicate mean values and standard deviations; statistical significance levels were calculated by Student’s *t*-test; the numbers of independent experiments are given in the Results section or Figure Legends. Final assembly and preparation of all figures for publication was done using Corel Draw Graphics Suite X4.

## Results

### CRN5 expression is up-regulated in colon carcinoma

**Fig. 1:**
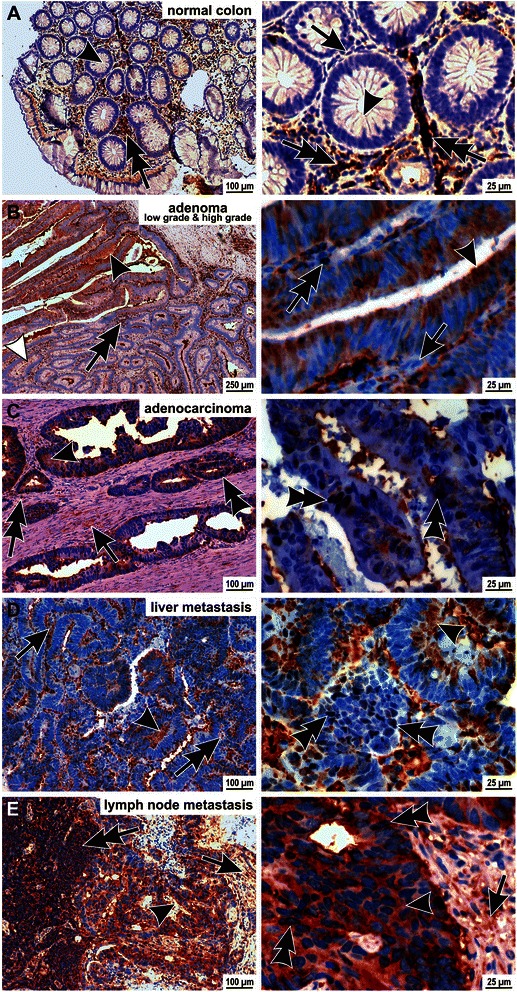
CRN5 is over-expressed in human colon cancer and is present in the nucleus of a subset of tumour cells. **a** Transverse section of normal human colon tissue. Colon epithelium cells (*arrowheads*) and fibroblasts of the stroma (*arrow*) did not express CRN5, while tissue-resident lymphocytes exhibited a marked expression of CRN5 (*double-arrows*). **b** Section of an adenoma with a low grade (*lower right region*) and a high grade part (*upper left region*). In the more benign part only single epithelial cells were faintly positive for CRN5 (*white arrowhead*). In contrast, all epithelial cells of the high grade part showed a strong cytosolic stain of CRN5 (*arrowheads*). In both parts stroma fibroblasts were CRN5-negative (*arrow*), and lymphocytes displayed the marked CRN5 signal (*double-arrows*). **c** Section of an adenocarcinoma with virtually all tumour epithelial cells showing a strong cytosolic CRN5 expression (*arrowhead*). Furthermore, a signal for CRN5 was present in the nuclei of tumour epithelial cells (*double-arrowheads*). In addition to the lymphocytes (*double-arrow*), stroma fibroblasts (*arrow*) were CRN5-positive in the adenocarcinoma. **d**, **e** Sections of liver and lymph node metastases, where a strong CRN5 signal intensity was visible in the cytoplasm (*arrowheads*) and nuclei (*double-arrowheads*) of the tumour epithelial cells. Tumour stroma cells (*arrows*) and lymphocytes (*double-arrows*) also showed CRN5 expression. The images at higher magnification (*right column*) were taken from the same section except for the high grade adenoma, which was from another tissue specimen

Expression of CRN5 in human colon tumours was investigated by histopathological analyses using our mouse monoclonal antibody K77-578-1. In normal colon tissue neither epithelial nor stroma cells did express CRN5, while tissue-residing lymphocytes showed a strong cytosolic localisation of CRN5 (Fig. 1a; intense reddish stain marked by double-arrows). In low grade adenoma few epithelial cells were detected, which expressed CRN5 in the cytosol. Upon further de-differentiation into high grade adenoma virtually all tumour epithelial cells were positive for CRN5 (Fig. 1b). In adenocarcinoma, CRN5 was additionally present in the nuclei of a subset of the tumour epithelial cells. Moreover, fibroblasts of the tumour stroma were also positive for CRN5 (Fig. 1c). This pattern of CRN5 expression in both tumour epithelial and stroma cells was also visible in liver and lymph node metastases (Fig. 1d, e). Semi-quantitative analysis showed that the tumour epithelial cells in all tissue specimens of high grade adenoma, carcinoma and metastases were CRN5 positive. In contrast, only in one third of the low grade adenomas CRN5 expression was found, while normal colon epithelial cells were negative for CRN5 (Additional file 1: Figure S1A). CRN5-positive stroma cells were restricted to colon carcinoma and metastases. Presence of CRN5 in nuclei of tumour epithelial cells was only evident in carcinoma and metastases (Additional file 1: Figure S1B).

Two antibodies, our mouse mAb K77-578-1 and the commercially available rabbit pAb L-20, were used for immunoblotting in order to compare the expression levels of CRN5 in colon tissue specimens. Both antibodies showed that the CRN5 signal intensity did not increase in colon carcinoma as compared to normal human colon tissue (Additional file 2: Figure S2A, B, left parts). This finding did, however, not contradict the CRN5 expression pattern seen by immunohistochemistry, as in the presence of an already strong expression of CRN5 in tissue-residing lymphocytes in the normal colon tissue (Fig. 1a, left image, double-arrow), the additional expression of CRN5 in colon epithelial tumour cells did not lead to an obvious increase of the overall CRN5 signal intensity in immunoblotting. Both antibodies detected CRN5 predominantly as a band of 48 kDa in normal colon and colon carcinoma tissues (Additional file 2: Figure S2A, B, left parts). This band was the exclusive signal of CRN5 in the WiDr and DLD-1 human colon carcinoma cell lines (Additional file 2: Figure S2A, B, right parts); and also murine CT26 and CMT93 and rat DHD/K12/TRb colon carcinoma cell lines solely showed this 48 kDa band (data not shown). In murine colon and non-colon cell lines (Additional file 2: Figure S2C, D), however, our own mouse mAb K77-578-1 and the three commercially available antibodies, i.e. goat pAb L-20, rabbit pAb H-110 (not shown), and mouse mAb A-3, detected CRN5 at the expected position of 56 kDa (calculated molecular mass 59.8 kDa). Specificity of our mAb K77-578-1 was further verified by detection of a GFP-CRN5 fusion protein (Additional file 2: Figure S2D).

### CRN5 is present in nuclei, at F-actin stress fibres, and the front of lamellipodia

**Fig. 2:**
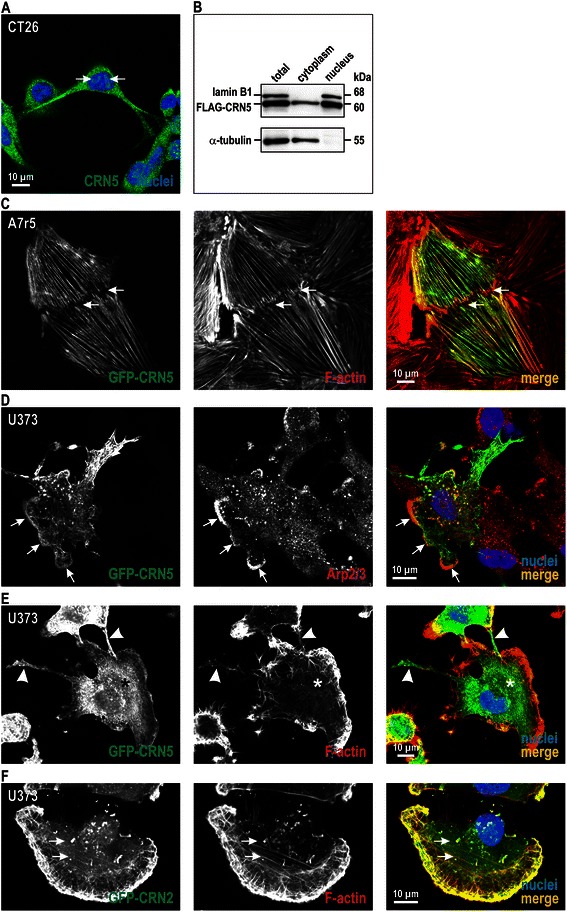
CRN5 localises to the nucleus, F-actin stress fibres, and lamellipodia. **a** Cytosolic and nuclear (*arrows*) localisation of CRN5 in CT26 mouse colon carcinoma cells. Detection was done with pAb H-110. Cells were fixed with 4 % paraformaldehyde, permeabilised with Triton X-100, and treated with 0.05 % Trypsin for 10 min for epitope retrieval according to [25]. Secondary antibody, goat anti-mouse AlexaFluor 488; nuclei were visualized with DAPI. **b** Nuclei of HeLa cells over-expressing FLAG-tagged CRN5 were isolated, and presence of CRN5 was determined by pAb H-110 immunoblotting. For control, the same membrane was labelled with lamin B1 (nuclear envelope) and α-tubulin (cytoplasm) antibodies. **c** In A7r5 aortic smooth muscle cells GFP-CRN5 localised to F-actin stress fibres; arrows label a cell-cell contact. F-actin was visualised using TRITC-phalloidin. **d** In U373 human glioblastoma cells GFP-CRN5 co-localised with the Arp2/3 complex (anti-p34 antibody) at the front of lamellipodia (*arrows*). **e**, **f** U373 human glioblastoma cells over-expressing GFP-CRN5 or GFP-CRN2. While GFP-CRN2 was strongly enriched at all F-actin structures including stress fibres, the presence of GFP-CRN5 led to depletion of F-actin stress fibres (*asterisk*) and induced extensions at the rear of the cells (*arrowheads*)

To address the re-localisation of CRN5 into the nucleus, which we had seen in the colonic tissue specimens, we used CT26 mouse colon carcinoma cells. A nuclear localisation of endogenous CRN5 was also detected in these cells after epitope retrieval (Fig. 2a). Nuclear CRN5 was further verified by fractionation experiments using HeLa cells, which could easily be transfected to over-express FLAG-CRN5 (Fig. 2b). We also studied the cytosolic localisation of CRN5 in more detail, as a previous study suggested the exclusive presence of CRN5-GFP at focal adhesions [11]. We found that the localisation of CRN5 was strongly dependent on the cell type. While GFP-CRN5 indeed co-localised with vinculin at focal adhesions in HeLa human cervical cancer cells (data not shown), it was also present at other actin structures, for example at F-actin stress fibres in A7r5 rat smooth muscle cells and the front of lamellipodia in U373 human glioblastoma cells (Fig. 2c, d). Moreover, we noted that stable over-expression of GFP-CRN5 in U373 cells almost completely suppressed F-actin stress fibres and caused the formation of multiple long extensions enriched in GFP-CRN5 at the rear of the cells (Fig. 2e). These effects were specific for CRN5, as they could not be observed in U373 cells over-expressing the coronin paralog CRN2 (Fig. 2f).

### CRN5 expression level correlates with cell migration velocity

**Fig. 3:**
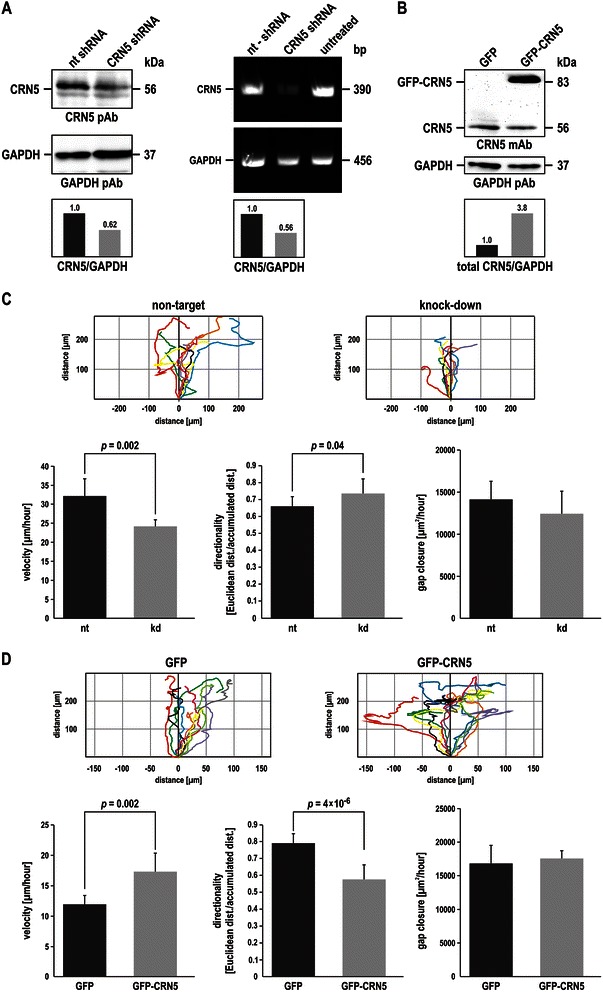
Expression of CRN5 increases cell migration velocity. **a** Lentivirus-mediated expression of a CRN5-specific shRNA caused an approximately 40 % reduction of both the CRN5 mRNA and protein levels in CT26 mouse colon carcinoma cells. A representative immunoblot and agarose gel as well as corresponding densitometry values are shown. CT26 control cells expressed a non-target (nt) shRNA. **b** Lentivirus-mediated GFP-CRN5 over-expression in U373 human glioblastoma cells led to a 3.8-fold increase of the total CRN5 protein level as compared to control cells expressing only GFP. A representative immunoblot as well as densitometry values are shown. **c** CT26 cells expressing the non-target or CRN5-specific shRNA (knock-down, kd) and (**d**) U373 cells over-expressing GFP or GFP-CRN5 were used for analysis of cell migration employing *in vitro* wound healing assays. Plot graphs show two-dimensional migration patterns of individual cells. Column charts indicate mean values and standard deviations of migration velocity and directionality (CT26 cell lines, n = 96 cells each; U373 cell lines, n = 150 cells each) as well as wound closure (CT26 cell lines, n = 6 gaps each; U373 cell lines, n = 10 gaps each)

To further analyse the role of CRN5 in tumour cell migration, we aimed at knocking down CRN5 in cell lines derived from human colon carcinoma. We used a lentiviral transduction protocol to express shRNA oligos directed against CRN5 in human WiDr and DLD-1 cells. We tested 12 different shRNA oligos, but none of them had a significant effect on the CRN5 expression level, although transduction and expression of a non-target shRNA worked. Two of the shRNAs we used targeted sequence stretches of CRN5 paralogous to sequences successfully used for knock-down of CRN2 [27]. Using a commercially available siRNA pool (Thermo Scientific Dharmacon, containing siRNAs J-011420-05, J-011420-06, J-011420-07, J-011420-08) also did not lead to a CRN5 knock-down. We therefore changed to murine and rat cell lines and employed murine CT26 and CMT93 and rat DHD/K12KTRb colon carcinoma cells for further tests of CRN5-specific shRNAs. Using a shRNA that targets the sequence GGAACGTCTTGGACATCAT, which is comparable to the one used for RAW264.7 murine macrophages [13] and identical, but independently designed, to the one used for MTLn3 rat mammary adenocarcinoma cells [11], we finally could generate a knock-down of CRN5, however, only in CT26 cells. This CRN5-specific shRNA led to a moderate reduction of CRN5 expression of approximately 40 % both at the mRNA and protein level (Fig. 3a). These cells were subjected to cell migration and *in vitro* wound healing assays. Analysis of the CT26 non-target shRNA control cells showed a mean migration velocity of 32.1 μm/h and a directionality of 0.66. Knock-down of CRN5 resulted in a significantly reduced migration velocity of 24.1 μm/h and an increased directionality of 0.74. In addition, the overall gap closure was decreased in the knock-down cells, but did not reach statistical significance (Fig. 3c).

When we next tried to over-express CRN5 in the above cell lines, we encountered similar difficulties. Neither by means of transfection nor lentiviral transduction we were able to generate any colon carcinoma cell line with stable over-expression of GFP-CRN5. However, U373 human glioblastoma cells, which we used as a control for the lentiviral transduction procedure, eventually showed a stable GFP-CRN5 over-expression (Fig. 3b). Analysis of these cells showed that U373 cells expressing GFP for control migrated with a mean velocity of 11.9 μm/h and a directionality of 0.8. In contrast, GFP-CRN5 over-expressing U373 cells migrated with an increased velocity of 17.3 μm/h and a decreased directionality of 0.5; the overall gap closure was not significantly changed (Fig. 3d). Although CT26 and U373 cells displayed different basal values for migration, the overall effect of a higher CRN5 expression level was a faster cell migration in conjunction with reduced directionality, while a decrease in CRN5 expression had the opposite effect.

### MAPK14 phosphorylates CRN5 at S423

**Fig. 4:**
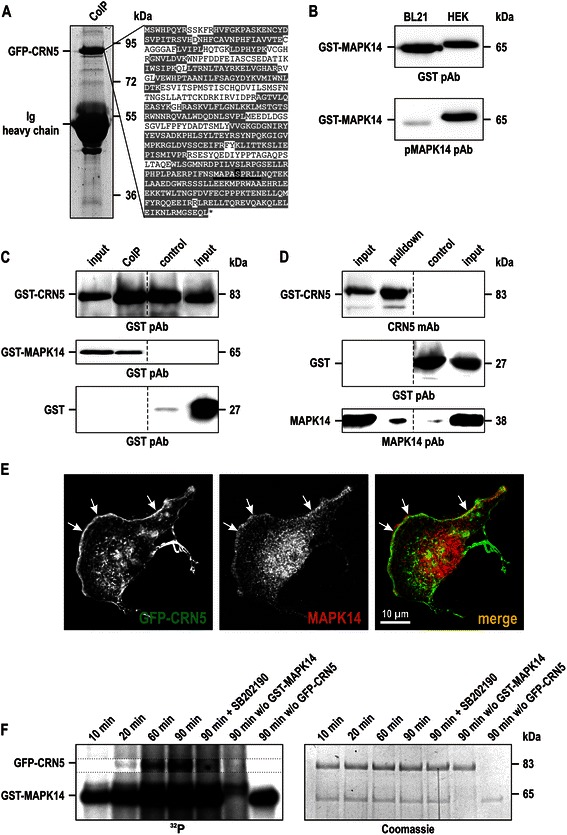
MAPK14 phosphorylates CRN5 at serine 423. **a** GFP-CRN5 was immunoprecipitated from lysates of U373 cells using mAb K77-578-1. The GFP-CRN5 band at 83 kDa was cut from the Coomassie stained gel, subjected to mass spectrometry, and a phosphorylation was identified at serine 423 (*highlighted in black*). Underlined, MAPK14 phosphorylation consensus target motif. Amino acid sequences highlighted in grey indicate the sequence coverage by mass spectrometry. **b** GST-MAPK14 expressed in *E. coli* BL21 and HEK293 cells. MAPK14 derived from the latter is phosphorylated at Thr180 and Tyr182 as determined by phospho-MAPK14 pAb D3F9, and is visible at a slightly higher molecular weight than the non-phosphorylated protein derived from the bacteria. **c** Co-immunoprecipitation studies were carried out using the CRN5 mAb K77-578-1 coupled to Protein G-coated magnetic microbeads (Miltenyi), which were first incubated with GST-CRN5 purified from *E. coli* BL21 and afterwards incubated with either GST-MAPK14 purified from HEK293 cells or GST purified from *E. coli* BL21 for control. Analysis was done by GST rabbit pAb [21] immunoblotting. **d** A pull-down assay was performed using GST-CRN5 or GST coated glutathione-sepharose beads together with non-tagged MAPK14. All three recombinant proteins were purified from *E. coli* BL21 as GST-fusion proteins; from MAPK14 the tag was cleaved by TEV-protease. Analysis was performed using CRN5 mAb K77-578-1, MAPK14 rabbit pAb, and the GST pAb. **c**, **d** For illustration purposes the order of lanes from the original immunoblots were digitally re-arranged to omit dispensable lanes. **e** Immunofluorescence images show an enrichment and co-localization of CRN5 and MAPK14 at the front of lamellipodia (*arrows*). U373 cells stably over-expressing GFP-CRN5 were methanol fixed; detection of MAPK14 was done with pAb #9212 and secondary antibody goat anti-mouse AlexaFluor 568. **f** *In vitro* kinase assay employing full-length GFP-CRN5 and GST-tagged MAPK14 purified from HEK293 cells. Time course (10, 20, 60, 90 min) of phosphate incorporation from [γ-^32^P]ATP into GFP-CRN5. As controls the selective, ATP-competitive MAPK14 kinase inhibitor SB202190 was used, and samples lacking GST-MAPK14 or GFP-CRN5. Left panel, autoradiograph (^32^P); right panel, corresponding Coomassie brilliant blue stained gel. Note, that the part of the autoradiograph showing the time-dependent phosphorylation of GFP-CRN5 is presented with increased contrast settings (*dotted rectangle*), because of the high background signals from MAPK14 autophosphorylation

We performed co-immunoprecipitation and pull-down experiments in order to identify novel, tumour-related post-translational modifications and binding partners of CRN5. Mass spectrometry analyses of GFP-CRN5 immunoprecipitated from U373 cell lysates revealed phosphorylation of serine 423 (Fig. 4a). This residue is located within the C-terminal extension of CRN5 and is part of a MAPK14 target consensus motif [31]. To investigate a possible direct interaction between both proteins, pull-down assays were carried out with recombinant purified MAPK14 isolated either from *E. coli* BL21 or HEK293 cells in conjunction with bacterially expressed GST-CRN5. Note that MAPK14 derived from mammalian cells is phosphorylated and active, whereas the bacterially expressed protein is non-phosphorylated and enzymatically inactive (Fig. 4b). GST-CRN5 was pre-incubated with CRN5 mAb K77-578-1 coupled to Protein G-coated magnetic microbeads and then incubated with GST-MAPK14 or GST for control, both purified from HEK293 cells (Fig. 4c). Similarly, MAPK14 purified from *E. coli* was incubated with GST-CRN5 or GST coupled to glutathione-sepharose beads (Fig. 4d). These experiments showed that CRN5 directly binds to both non-phosphorylated and phosphorylated MAPK14. At the cellular level, we observed a co-localisation of both proteins at the front of lamellipodia (Fig. 4e). Finally, we performed *in vitro* kinase assays to verify that CRN5 is a substrate of MAPK14. Incubation of both proteins led to a time-dependent increase of the CRN5 phosphorylation signal. Specificity of the reaction was confirmed using the ATP-competitive MAPK14 kinase inhibitor SB202190, which markedly suppressed CRN5 phosphorylation (Fig. 4f).

### Over-expression of CRN5 activates the MAPK14 signalling pathway

**Fig. 5:**
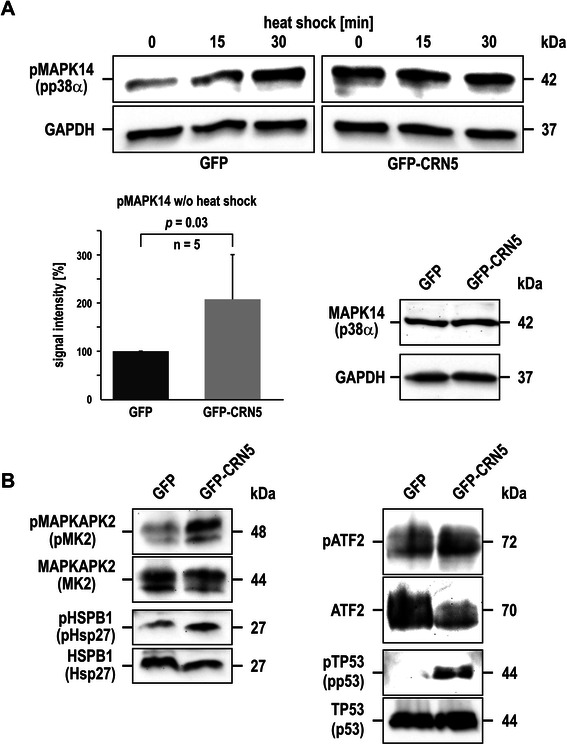
Over-expression of CRN5 activates the MAPK14 signalling pathway. **a** HEK293 cells stably over-expressing GFP for control (*left upper panel*) or GFP-CRN5 (*right upper panel*) were exposed to heat shock (0, 15, 30 min at 42 °C). Levels of Thr180/Tyr182-phosphorylated MAPK14 (pp38α) were determined by immunoblotting. Total MAPK14 (p38α, time point 0 min/without heat shock) and GAPDH of the heat shock experiment are shown for control (*right lower panel*). Densitometric analysis from five independent assays at time point 0 without heat shock (column chart with mean values and standard deviations; values were normalised to the GAPDH signals and indexed to GFP cells) revealed a two-fold increase of MAPK14 phosphorylation in cells expressing GFP-CRN5. **b** Analysis of the phosphorylation level of selected substrates downstream of MAPK14 revealed an activation of the MAPK14 pathway in GFP-CRN5 expressing HEK293 cells (Thr334-phosphorylated MAPKAPK2, Ser82-phosphorylated Hsp27, Thr71-phosphorylated ATF2, Ser392-phosphorylated p53). Loading control, see GAPDH in (**a**, *right lower panel*)

We next investigated whether CRN5 plays a role in MAPK14 activation. For this purpose we used a set of antibodies to determine the amounts of total and phosphorylated MAPK14, downstream kinases, and substrates. We used HEK293 cells for these experiments, because heat shock treatment of the control cells expressing GFP resulted in a clear, time-dependent increase of MAPK14 phosphorylation. In contrast, HEK293 cells that over-expressed GFP-CRN5 already showed a two-fold increased MAPK14 phosphorylation at basal conditions with no further increase upon heat shock stimulation, and the total MAPK14 content did not change (Fig. 5a). Furthermore, the downstream kinase MAPKAPK2 as well as the cytosolic (Hsp27) and nuclear (activating transcription factor (ATF2) p53) substrates of MAPK14 showed increased phosphorylation levels in the GFP-CRN5 over-expressing cells (Fig. 5b).

### CRN5 is a substrate of PRMT5 and increases TRAIL receptor sensitivity

Our co-immunoprecipitation and pull-down experiments also led to the identification of the 72 kDa protein PRMT5 (protein arginine N-methyltransferase 5) as a novel, direct CRN5 binding partner (Additional file 3: Figure S3A, B). We performed ^3^H-labelled S-adenosylmethionine *in vitro* methylation assays and found that CRN5 is a substrate of PRMT5 (Additional file 3: Figure S3C). In a next step, we investigated the effect of CRN5 on TRAIL (TNF-Related Apoptosis-Inducing Ligand)-induced cell death, as a previous study had identified PRMT5 as a novel TRAIL receptor-binding protein, whose knock-down restored the sensitivity of several tumour cell lines to TRAIL-induced cell death [19]. We used HeLa cells, a well-established TRAIL-resistant cell line [32], to investigate the effect of CRN5 over-expression with and without additional PRMT5 knock-down on the TRAIL receptor pathway (Additional file 3: Figure S3D; note, that HeLa cells virtually lack endogenous CRN5). Knock-down of PRMT5 had no effect on cell proliferation in absence of TRAIL, while expression of FLAG-CRN5 increased cell proliferation by approximately 20 % (Additional file 3: Figure S3E, dark columns). Although in our experiments the addition of TRAIL ligand to HeLa cells control-transfected with empty vectors already caused a 40 % reduction of the cell number, knock-down of PRMT5 resulted in a further 10 % decrease (Additional file 3: Figure S3E, light columns left and right panels). Both, over-expression of FLAG-CRN5 alone and in combination with PRMT5 knock-down reduced the cell number by approximately 20 % (Additional file 3: Figure S3E, light columns right panel).

## Discussion

Our CRN5 immunohistochemistry analysis revealed an increase of the CRN5 expression level in colon tumour cells during adenoma-adenocarcinoma progression. In addition, our cell migration experiments showed that a higher CRN5 expression level causes an increased tumour cell migration velocity. Although this is the first report on a putative function of CRN5 in the malignant progression of cancer, it seems to be a general pathophysiological feature of coronin proteins. Previous studies reported an involvement of the paralog CRN2 (synonyms are: Coro1C, coronin 3, hCRNN4) in multiple types of cancer. In human diffuse gliomas the number of CRN2 positive tumour cells was correlated with the malignant phenotype of diffuse gliomas [27], and an increased CRN2 expression level promoted glioblastoma cell invasion [28]. Over-expression of CRN2 also enhanced cell migration in BEL-7402 human hepatocellular carcinoma [33] and in MKN28-NM gastric cancer cells [34]. Other studies showed increased CRN2 protein expression related to progression of dysplastic naevi [35], the clinical progression and pulmonary metastasis of hepatocellular carcinoma [36], the pathogenesis of primary effusion lymphoma [37], and the presence of lymph node metastases in gastric cancer [34]. Increased CRN2 mRNA levels were correlated to further tumour types with poor clinical prognosis [38–41]. For another coronin paralog, CRN4 (synonyms are: coronin 1A, coronin 1, ClipinA, Taco, p57), published data indicated increased expression levels in breast cancer [42–44].

A role of coronin proteins in tumour progression is not surprising, as basic characteristics of various coronin proteins are their interaction with actin polymers as well as the regulation of actin cytoskeleton dynamics [6]. Generally, the actin cytoskeleton is highly important for the processes of tumour cell migration and invasion [45]. In this respect, we found that CRN5 not only localises to F-actin enriched cell compartments including F-actin stress fibres, the front of lamellipodia and focal adhesions, but also modulates the actin cytoskeleton by reducing the number of F-actin stress fibres leading to an elevated cell migration velocity. However, coronin proteins do not only regulate actin dynamics via direct interaction with F-actin, but also by modulation of related signalling pathways [46–50]. The finding that CRN4 is involved in calcium homeostasis [51, 52] and CRN5 in the expression of inflammatory response genes via NCoR complex clearance in immune cells [13] fulfils the expectation that the β-propeller fold of coronin proteins serves as platform for multiple protein-protein interactions. We therefore have searched for novel and tumour related CRN5 binding partners by co-immunoprecipitation experiments, mass spectrometry analyses, and pull-down assays. Amongst several newly identified proteins MAPK14 and PRMT5 were related to tumourigenesis. Pharmacological inhibition of MAPK14 inhibited growth of colon cancer cell lines [17] and colon tumour growth in mice [15]. In our experiments the interaction of CRN5 with MAPK14 led to phosphorylation of CRN5. In addition, cells that over-expressed CRN5 showed an activation of the MAPK14 pathway suggesting an involvement of CRN5 in tumour progression via the MAPK14 signalling pathway. With regard to PRMT5 it has been reported that a knock-down of this methyl transferase restored the sensitivity of several tumour cell lines to TRAIL-induced cell death [19]. We found that CRN5 is methylated by PRMT5 and that over-expression of CRN5 also increased the sensitivity to TRAIL-induced cell death. This latter, rather anti-oncogenic effect might become relevant in conjunction with cancer treatment.

Taken together, our present work links the expression level of CRN5 with the malignant progression of colon cancer. Further, our results suggest that CRN5 affects tumour-related cell functions on several levels, directly on the actin cytoskeleton and more indirectly via modulation of the MAPK14 and PRMT5 signalling pathways.

## Conclusions

This is the first time it has been reported that increased expression of coronin 2A/CRN5 is associated with the colorectal adenoma-adenocarcinoma progression. Over-expression of CRN5 does not only induce cytoskeletal alterations, but also changes of the MAPK14 and PRMT5 signalling pathways. The present study adds further evidence that members of the coronin family of actin regulatory proteins play important roles in cancer progression.

## Additional files

Additional file 1: Figure S1. Expression level and nuclear localisation of CRN5 are associated with the malignancy of colon tumours. (**A**) Semi-quantitative analysis (per cent values) of the expression of CRN5 in normal and tumour epithelial cells, stroma cells, and lymphocytes as seen in Fig. 1. (**B**) Semi-quantitative analysis (per cent values) of the presence of CRN5 in the cytoplasm or nuclei of tumour epithelial cells as seen in Fig. 1. N, number of diagnostic tissue specimens. Asterisk, only two of the six specimens from low grade adenoma showed CRN5 expression in tumour epithelial cells. (TIFF 225 kb)
Additional file 2: Figure S2. CRN5 immunoblotting indicates two bands with molecular weights of 56 and 48 kDa. The immunoblotting pattern of mouse mAb K77-578-1 was compared with the commercially available CRN5 antibodies L-20 and A-3. The antibodies detected endogenous CRN5 at 56 and 48 kDa in human colon tissues; total protein amounts of the tissue lysates were determined and identical amounts loaded onto the gel. Specificity of mAb K77-578-1 was further verified by detection of a GFP-CRN5 fusion protein. For illustration purposes lanes from different original immunoblots were digitally combined. Hs, homo sapiens; Mm, mus musculus. (TIFF 277 kb)
Additional file 3: Figure S3. CRN5 is methylated by PRMT5, increases cell proliferation, and sensitises cells to TRAIL-induced cell death. (**A**) Co-immunoprecipitation experiment using lysates from U373 cells with stable over-expression of GFP-CRN5 and anti-GFP magnetic microbeads. Precipitates were subjected to SDS-PAGE, stained with Coomassie brilliant blue, and proteins were identified by mass spectrometry analysis. Arrows indicate the positions of GFP-CRN5 and PRMT5. (**B**) Pull-down assay using GST-CRN5 or GST for control coupled to glutathione-coated sepharose beads and soluble His6-PRMT5 confirm a direct interaction of CRN5 and PRMT5. Proteins were expressed and purified from *E. coli* strain BL21. Asterisk, GST signal from partial degradation of GST-CRN5. For illustration purposes the order of lanes from the original immunoblots were digitally re-arranged to omit dispensable lanes. (**C**) *In vitro* methylation experiments based on the incubation of recombinant PRMT5/MEP50 complex, GFP-CRN5, and ^3^H-labelled S-adenosylmethionine showed methylation of CRN5. Controls lacked the substrate GFP-CRN5. Columns indicate mean values and standard deviations derived from three independent experiments. (**D**) Immunoblot verifications of PRMT5 knock-down and FLAG-CRN5 over-expression in HeLa cells. (**E**) Left panel: HeLa cell viability after double-transfections as indicated in absence (dark columns) and presence (light columns) of TRAIL ligand. Viability of control cells transfected with both empty vectors is set to 100 %. One representative out of ten independent experiments with similar patterns of cell viability is shown; note, that the level of absolute absorbance values varied from experiment to experiment and did not allow calculation of a mean value. Right panel: For better illustration of the results the three light columns indicate the decrease of cell viability, i.e. the rate of TRAIL-induced cell death. This was calculated by the differences of the values from the three right pairs of dark and light bars from the left panel minus the TRAIL-induced decrease of cell viability of the control cells (first left pair of bars). (TIFF 521 kb)

## Abbreviations

AdOx: Adenosine dialdehyde
AEC: 3-amino-9-ethylcarbazole
ATCC: American type culture collection
BSA: Bovine serum albumin
CRN: Coronin
DTT: Dithiothreitol
ECACC: European collection of cell cultures
EDTA: Ethylenediaminetetraacetic acid
EGFP: Enhanced green fluorescent protein
EGTA: Ethylene glycol tetraacetic acid
ESI: Electrospray ionization
FBS: Foetal bovine serum
GAPDH: Glyceraldehyde 3-phosphate dehydrogenase
GST: Glutathione S-transferase
LXR: Liver X receptor
MAPK14: Mitogen activated protein kinase 14 MAP kinase p38α
NCoR: Nuclear receptor co-repressor
NP-40: Octylphenoxypolyethoxylethanol Nonidet P-40, IGEPAL CA-630
PBS: Phosphate buffered saline
PMSF: Phenylmethylsulfonyl fluoride
PRMT5: Protein arginine N-methyltransferase 5
PVDF: Polyvinylidene difluoride
SDS-PAGE: Sodium dodecyl sulfate polyacrylamide gel electrophoresis
shRNA: Small hairpin ribonucleic acid
siRNA: Small interfering ribonucleic acid
SUMO: Small ubiquitin-like modifier
TBS-T: Tris-buffered saline with Tween 20 detergent
TEV: Tobacco etch virus
TRAIL: Tumour necrosis factor related apoptosis inducing ligand
