# Supplementary figures and images for: Coronin 2A (CRN5) expression is associated with colorectal adenoma-adenocarcinoma sequence and oncogenic signalling
Source: BMC Cancer. 2015 Sep 15;15:638. doi: 10.1186/s12885-015-1645-7 (PMC4612562; doi:10.1186/s12885-015-1645-7)

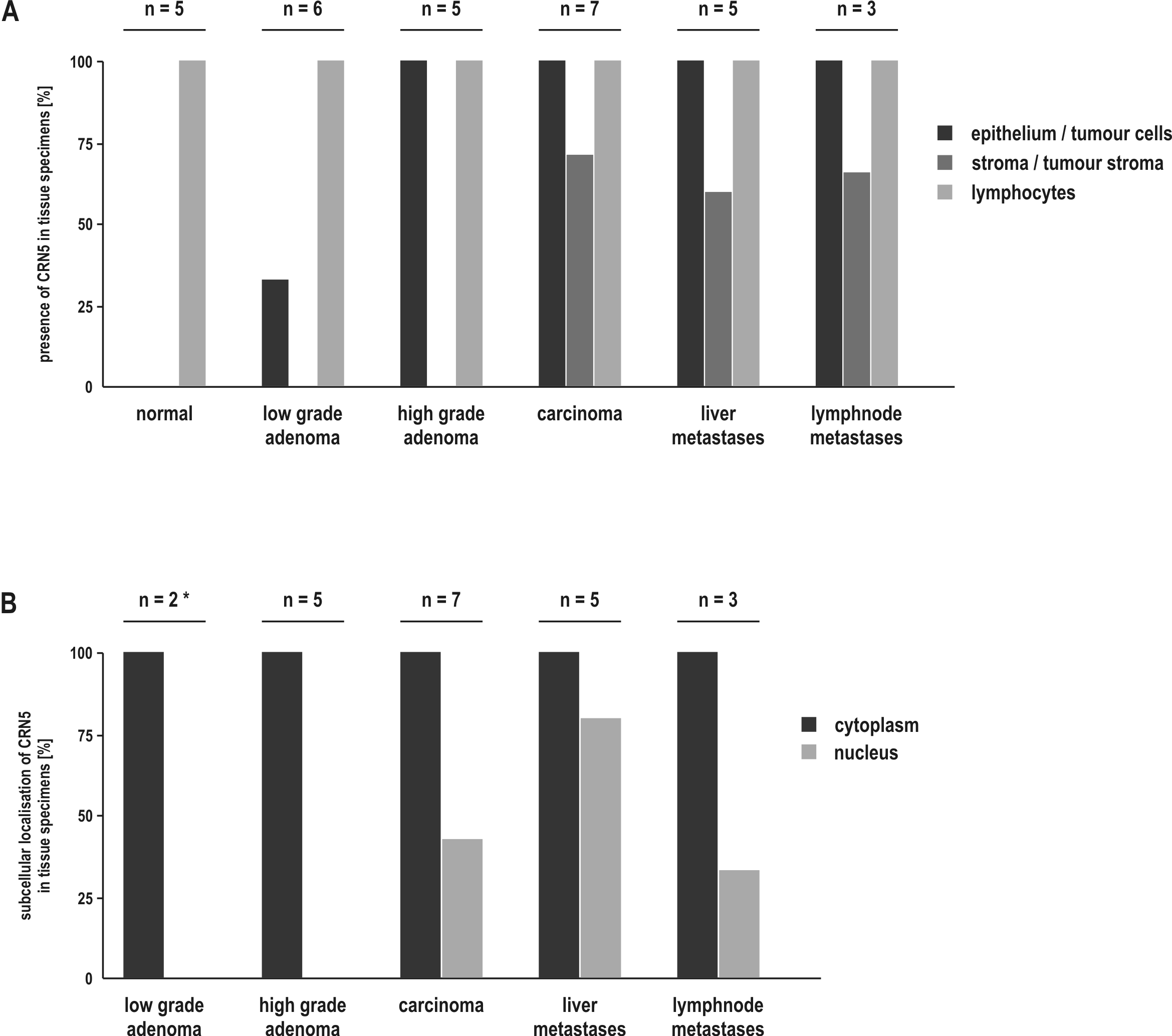

Supplement: Additional file 1: Figure S1. — Expression level and nuclear localisation of CRN5 are associated with the malignancy of colon tumours. (A) Semi-quantitative analysis (per cent values) of the expression of CRN5 in normal and tumour epithelial cells, stroma cells, and lymphocytes as seen in Fig. 1. (B) Semi-quantitative analysis (per cent values) of the presence of CRN5 in the cytoplasm or nuclei of tumour epithelial cells as seen in Fig. 1. N, number of diagnostic tissue specimens. Asterisk, only two of the six specimens from low grade adenoma showed CRN5 expression in tumour epithelial cells. (TIFF 225 kb) [file 12885_2015_1645_MOESM1_ESM.tiff]

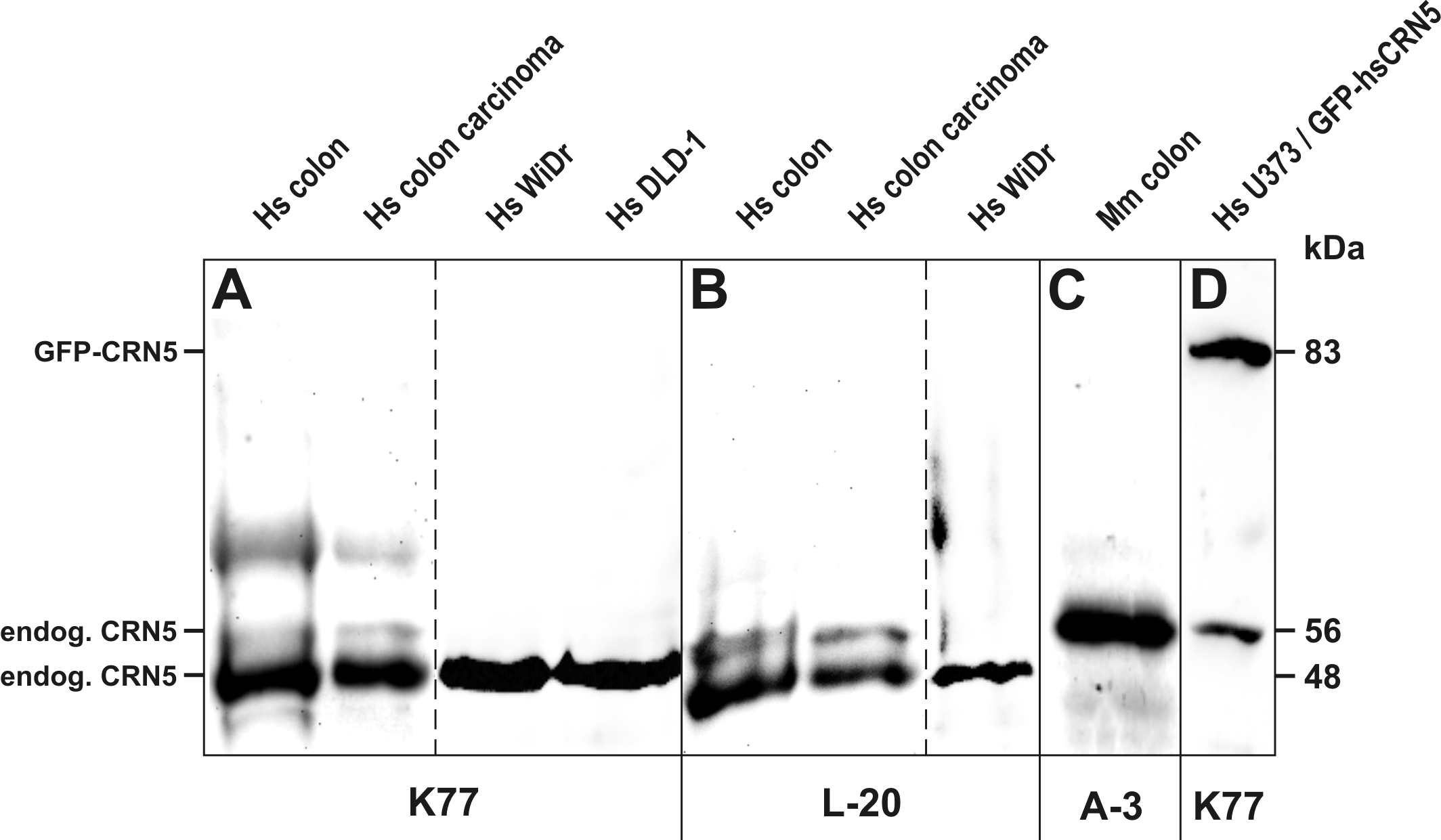

Supplement: Additional file 2: Figure S2. — CRN5 immunoblotting indicates two bands with molecular weights of 56 and 48 kDa. The immunoblotting pattern of mouse mAb K77-578-1 was compared with the commercially available CRN5 antibodies L-20 and A-3. The antibodies detected endogenous CRN5 at 56 and 48 kDa in human colon tissues; total protein amounts of the tissue lysates were determined and identical amounts loaded onto the gel. Specificity of mAb K77-578-1 was further verified by detection of a GFP-CRN5 fusion protein. For illustration purposes lanes from different original immunoblots were digitally combined. Hs, homo sapiens; Mm, mus musculus. (TIFF 277 kb) [file 12885_2015_1645_MOESM2_ESM.tiff]

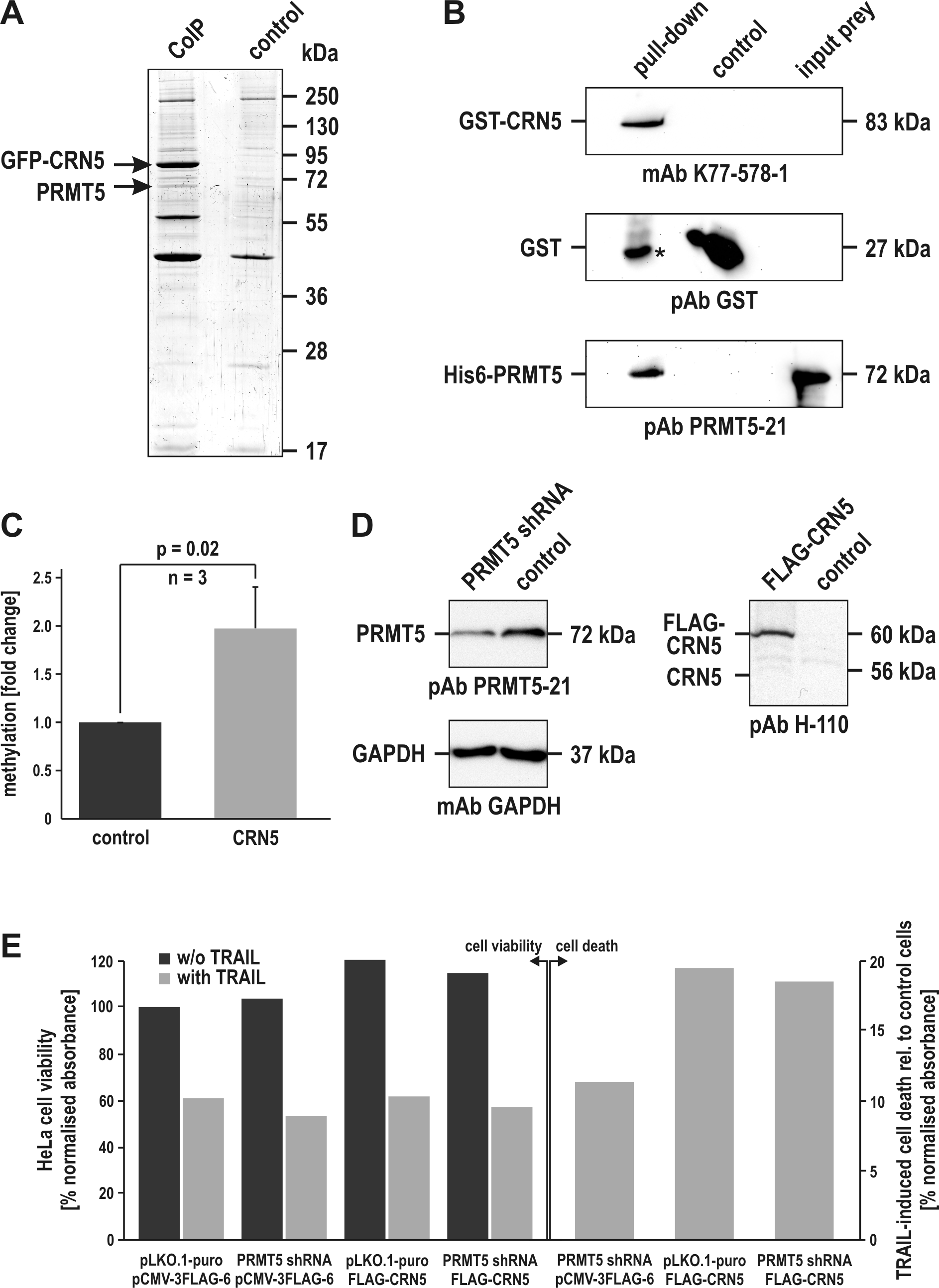

Supplement: Additional file 3: Figure S3. — CRN5 is methylated by PRMT5, increases cell proliferation, and sensitises cells to TRAIL-induced cell death. (A) Co-immunoprecipitation experiment using lysates from U373 cells with stable over-expression of GFP-CRN5 and anti-GFP magnetic microbeads. Precipitates were subjected to SDS-PAGE, stained with Coomassie brilliant blue, and proteins were identified by mass spectrometry analysis. Arrows indicate the positions of GFP-CRN5 and PRMT5. (B) Pull-down assay using GST-CRN5 or GST for control coupled to glutathione-coated sepharose beads and soluble His6-PRMT5 confirm a direct interaction of CRN5 and PRMT5. Proteins were expressed and purified from E. coli strain BL21. Asterisk, GST signal from partial degradation of GST-CRN5. For illustration purposes the order of lanes from the original immunoblots were digitally re-arranged to omit dispensable lanes. (C) In vitro methylation experiments based on the incubation of recombinant PRMT5/MEP50 complex, GFP-CRN5, and 3H-labelled S-adenosylmethionine showed methylation of CRN5. Controls lacked the substrate GFP-CRN5. Columns indicate mean values and standard deviations derived from three independent experiments. (D) Immunoblot verifications of PRMT5 knock-down and FLAG-CRN5 over-expression in HeLa cells. (E) Left panel: HeLa cell viability after double-transfections as indicated in absence (dark columns) and presence (light columns) of TRAIL ligand. Viability of control cells transfected with both empty vectors is set to 100 %. One representative out of ten independent experiments with similar patterns of cell viability is shown; note, that the level of absolute absorbance values varied from experiment to experiment and did not allow calculation of a mean value. Right panel: For better illustration of the results the three light columns indicate the decrease of cell viability, i.e. the rate of TRAIL-induced cell death. This was calculated by the differences of the values from the three right pairs of dark and light bars f [file 12885_2015_1645_MOESM3_ESM.tiff]
